# Histological Processing of CAD/CAM Titanium Scaffold after Long-Term Failure in Cranioplasty

**DOI:** 10.3390/ma15030982

**Published:** 2022-01-27

**Authors:** Heilwig Fischer, Claudius Steffen, Katharina Schmidt-Bleek, Georg N. Duda, Max Heiland, Carsten Rendenbach, Jan-Dirk Raguse

**Affiliations:** 1 Department of Oral and Maxillofacial Surgery, Charité-Universitätsmedizin, Augustenburger Platz 1, 13353 Berlin, Germany; heilwig.fischer@charite.de (H.F.); max.heiland@charite.de (M.H.); carsten.rendenbach@charite.de (C.R.); 2 Julius Wolff Institute and Berlin Institute of Health Center for Regenerative Therapies, Charité-Universitätsmedizin Berlin, Augustenburger Platz 1, 13353 Berlin, Germany; katharina.schmidt-bleek@charite.de (K.S.-B.); georg.duda@charite.de (G.N.D.); 3 Department of Oral and Maxillofacial Surgery, Fachklinik Hornheide, Dorbaumstraße 300, 48157 Münster, Germany; Jan-Dirk.Raguse@fachklinik-hornheide.de

**Keywords:** CAD/CAM, cranioplasty, bone regeneration, osteoconduction, scaffold

## Abstract

Cranioplasty is a frequently performed procedure after craniectomy and includes several techniques with different materials. Due to high overall complication rates, alloplastic implants are removed in many cases. Lack of implant material osseointegration is often assumed as a reason for failure, but no study has proven this in cranioplasty. This study histologically evaluates the osteointegration of a computer-aided design and computer-aided manufacturing (CAD/CAM) titanium scaffold with an open mesh structure used for cranioplasty. A CAD/CAM titanium scaffold was removed due to late soft tissue complications 7.6 years after cranioplasty. The histological analyses involved the preparation of non-decalcified slices from the scaffold’s inner and outer sides as well as a light-microscopic evaluation, including the quantification of the bone that had formed over the years. Within the scaffold pores, vital connective tissue with both blood vessels and nerves was found. Exclusive bone formation only occurred at the edges of the implant, covering 0.21% of the skin-facing outer surface area. The inner scaffold surface, facing towards the brain, did not show any mineralization at all. Although conventional alloplastic materials for cranioplasty reduce surgery time and provide good esthetic results while mechanically protecting the underlying structures, a lack of adequate stimuli could explain the limited bone formation found. CAD/CAM porous titanium scaffolds alone insufficiently osseointegrate in such large bone defects of the skull. Future research should investigate alternative routes that enable long-term osteointegration in order to reduce complication rates after cranioplasty. Opportunities could be found in mechano-biologically optimized scaffolds, material modifications, surface coatings, or other routes to sustain bone formation.

## 1. Introduction

To ensure patient survival in the management of elevated intracranial pressure (ICP) or herniation syndrome, craniectomies are carried out hundreds of times a day worldwide as an indispensable part of therapy [1]. There are various reasons for elevated ICP, including traumatic brain injury, intracranial hemorrhage, and infectious disease [1]. After surviving the initial event, reconstruction of the skull defect is required after craniectomy to ensure physical protection of the underlying brain, to reestablish cerebral fluid dynamics, and to restore the shape of the skull in esthetic terms [2]. The gold standard for bone defect reconstruction implies the reconstruction with autologous bone [3]. However, there are two main complications linked to the use of autologous bone that make the use of alloplastic material necessary, that is, bone resorption and infection [4]. Commonly used bone replacement materials in cranioplasty include titanium mesh, polymethyl methacrylate (PMMA), hydroxyapatite, and polyetheretherketone (PEEK) [2]. The ideal cranioplasty material should fit the following criteria: achieve a complete closure of the defect, radiolucency, resistance to infections, heat and mechanical stability, easy shaping, and low costs. However, no implant material exists which fulfills all of these criteria [5].

Titanium implants are described as the material of choice in secondary reconstructions in adults [6]. Custom-made titanium implants are reported as a reliable material with low long-term complication rates and favorable properties over other implants [7,8]. Their excellent osseointegration has been described in other parts of the body [9]. With computer-aided design and computer-aided manufacturing (CAD/CAM) techniques, they are also suitable for large defects [6]. The CAD/CAM technique allows the restoration of the initial shaping, and the use of scaffolds generally allows osseoinduction. This was demonstrated both in vitro and in animal experiments [9,10]. In humans, complete bony ingrowth into a scaffold has already been demonstrated in the spine [11]. However, in contrast to cranioplasty there is never any direct contact between the implant and the skin. Analyses of bony ingrowth in titanium scaffolds in cranioplasty do not exist. Nevertheless, ingrowth and overgrowth of bone into the scaffold is important to reduce complications. This indicates the importance of increased knowledge of this process in areas with high numbers of implant failure such as cranioplasty [4].

Despite the favorable characteristics of titanium implants for cranioplasty, late complications such as extrusion and infections are well known [12,13]. However, the reasons why these implants fail in the long term in cranioplasty remain unclear.

In this study, a CAD/CAM titanium scaffold that was used for cranioplasty of the left temporal region was removed due to late soft-tissue infection. This exemplary device provided the unique opportunity to improve our understanding of long-term failure in cranioplasty. No study has performed a detailed histological analysis of a removed implant for cranioplasty after long-term complications. Since ingrowth of bone into the scaffold is of importance to reduce long-term complications [4], we examined the scaffold concerning this aspect. We hypothesized that a lack of osseointegration of the implant is the reason for long-term complications such as extrusions or infections. The results of this study could form a basis for improvements of design and properties for bone formation at the implant side in cranioplasty.

## 2. Materials and Methods

### 2.1. Patient History and CAD/CAM Titanium Scaffold

**Figure 1:**
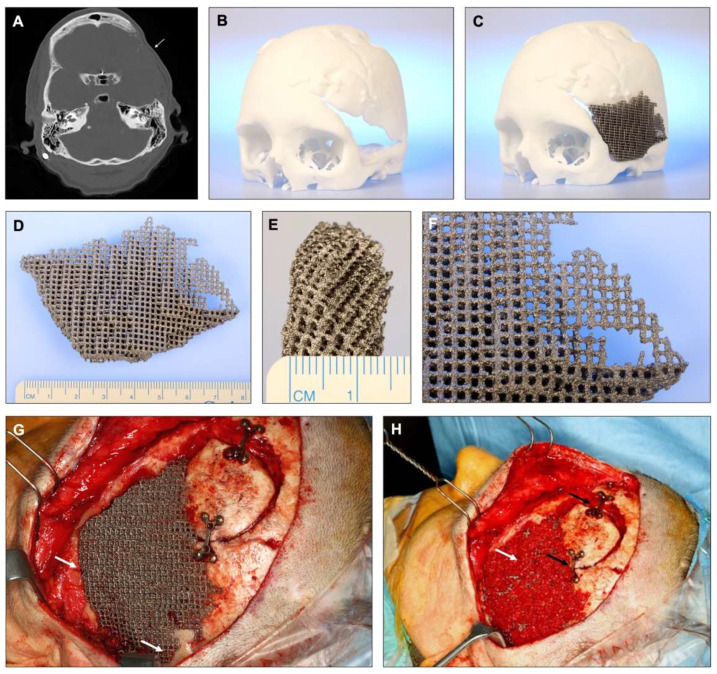
(**A**) Preoperative CT demonstrating the extensive bone defect (indicated by a white arrow) at the left frontotemporal region. (**B**) 3D-printed model of the patient’s skull, showing the triangular-shaped defect in the left frontotemporal region. (**C**) The computer-aided design and computer-aided manufacturing (CAD/CAM) titanium scaffold on the 3D-printed model of the patient’s skull. The CAD/CAM manufacturing allows the restoration of the bony outline in symmetry with the contralateral side. (**D**,**E**) Dimensions of the titanium scaffold. (**F**) Close up of the rough titanium surface showing an irregular structure. (**G**) Intraoperative situation immediately after reconstruction using the CAD/CAM titanium scaffold. Additional screws and a cerclage were used to fix the scaffold to the bone (white arrows). (**H**) titanium scaffold and overlaying iliac crest spongiosa (white arrow). A part of the defect could still be covered with autologous bone, fixed with two miniplates (black arrows).

Written informed consent was obtained from the patient for publication of this study and accompanying images. A 46-year-old female patient received a decompressive hemicraniectomy due to a Fisher grade 2 subarachnoid hemorrhage as a result of a ruptured anterior communicating artery aneurysm (7 × 4 × 5 mm). Reimplantation of the cryopreserved autologous bone flap was not successful due to resorption, leaving an osseous defect at the frontotemporal region of about 53 mm with an intact soft tissue situation (Figure 1).

Consequently, the procedure performed included a detachment of the temporal muscle from the bone, vitalizing of bony defect borders, and fixation of the scaffold using a cerclage and screws under general anesthesia. The procedure was performed one year after the initial event. Iliac crest spongiosa was placed onto the scaffold, the temporal muscle was used to cover the scaffold, and primary wound closure was performed.

At 7.6 years after reconstructive surgery, the patient presented again with an exposed temporal implant involving an area of 4 cm × 2 cm with perifocal infection and pus. The exposure was noticed by the patient 6 months before. The initial antibiotic treatment with ciprofloxacin administered due to infection with *Pseudomonas aeruginosa* was not successful. The removal of the adherent scaffold became necessary and further assessments of the scaffold were performed.

### 2.2. Histological Assessment

**Figure 2:**
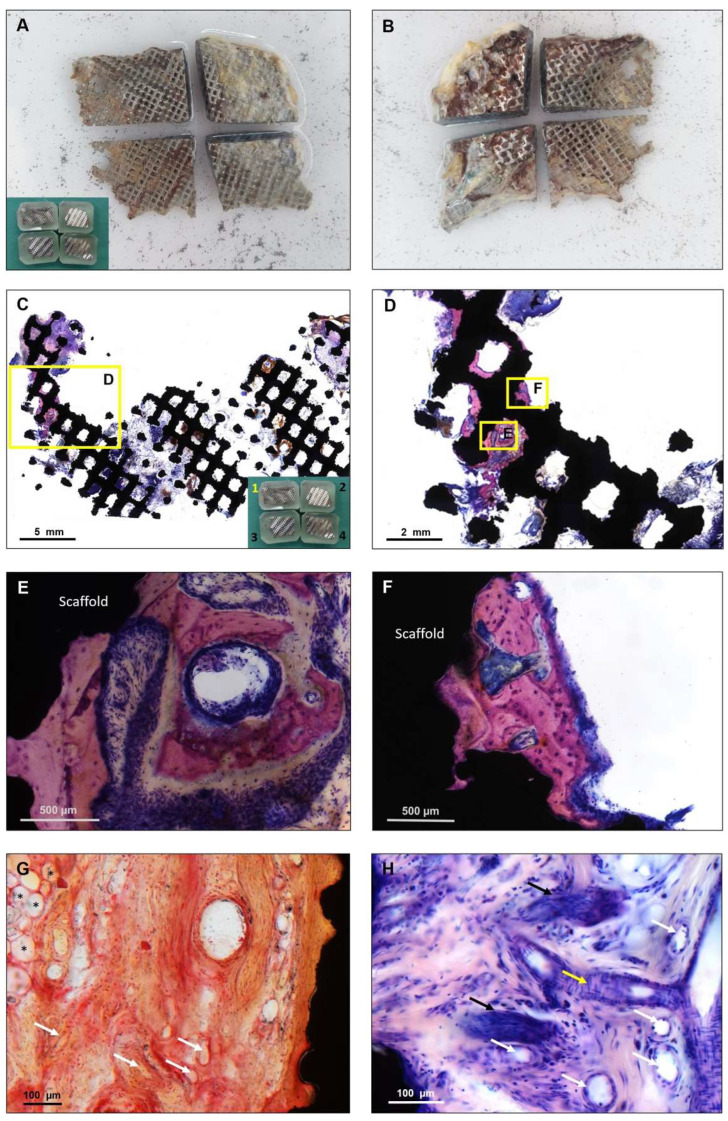
For histological embedding, the implant was cut twice to fit on the histological slides. (**A**) Inner surface of the cut implant; (**B**) outer surface. All attached tissue was left in place for embedding. In the bottom-left corner, the embedded parts can be seen in the four plastic blocks. After embedding, the plastic was ground down to the plane of the sample. The shiny silver surface of the embedded scaffold indicates the polished metal due to the grinding. For histological bone quantification, all samples were stained in Giemsa as an overview staining. The Giemsa stain shows mineralized bone in purple, and connective tissue in dark or light purple. (**C**) Part 1 section from the inner surface. Mineralized bone can be seen at the border as visible purple, growing in a very thin part of the scaffold (please compare to (A)). In the other parts of the scaffold, only connective tissue and inflammatory cells are present. In the patient, parts 2 and 4 were the frontal parts, oriented towards the patient’s face. (**D**) Zooming in on the bone formation, most of the purple-stained bone appears attached to the rough titanium surface. All bone formation appeared vital with clearly visible osteocytes (**E**,**F**). Between the bone and the scaffold, a direct and continuous contact surface is visible, serving as a sign of good osseointegration. (**G**) The Safranin O/Von Kossa staining reveals a high density in vascularization, with both visible arteries and veins (white arrows). In the absence of bone, connective tissue appeared in direct contact with the scaffold (black, on the right side of the image). On the left side, fat cells are visible (*). (**H**) A higher magnification of the Giemsa staining confirmed the findings from the Safranin O/Von Kossa staining. Diagonally (white arrows) and longitudinally (yellow arrow) cut vessels appeared embedded in connective tissue. In the bottom-left and upper-left corners, a black cross section of the scaffold is visible. Additionally, next to the vessels, nerves (marked with black arrows) were found.

In preparation for histological analysis, the specimen was fixed in formaldehyde for five days. After tissue fixation, the formaldehyde was washed out with water. The sample was subsequently dehydrated in an ascending alcohol series for 7 weeks to facilitate the penetration with the hydrophobic liquids of xylene and embedding material. Before embedding, the specimen was cut into four pieces (Figure 2A,B) using a diamond-coated saw (Exakt Diamant Band Saw Exakt 311 CL, Norderstedt, Germany) and then placed in xylene for 24 h to degrease the tissues and allow the infiltration with a plastic embedding material (Technovit 9100 New, Heraeus Kulzer, Hanau, Germany). After polymerization of the embedding material, a solid plastic block was formed. For further processing, the plastic was ground down to reach the plane of the sample. The preparation of non-decalcified slices allowed us to leave the titanium scaffold in the state in which it was explanted from the patient and to perform an evaluation of both the tissue and the titanium at the same time.

To allow a precise preparation of the non-decalcified sections, the samples were glued on microscopic slides from both sides. A slice of approximately 300 µm was cut off from each side of the block, using a diamond-coated saw to prepare slices from both the outer and the inner scaffold surface. In total, 8 slices were analyzed containing the whole scaffold area from both sides. The sample was then ground down to a thickness of 80 µm and polished (Präzisions-Mikro-Schleifsystem, Exakt, Norderstedt, Germany). Three samples were cut orthogonally to evaluate the cross section of the implant and prepared the same way. Staining was performed using a 10% Giemsa’s azur eosin methylene blue solution as an overview staining and for detecting mineralized bone.

The Giemsa stain was originally developed for smears and the Giemsa´s solution contains methylene blue as the main dye as well as eosin and Azure B. It is differentiated with xylene depending on the desired luminance of the colors. The Giemsa stain is considered a multiple stain because the different components of the tissue are dyed in different colors, not being the same colors as the ones from the mixture of dyes [14]. It provides a good color contrast between cells and the extracellular matrix of both hard and soft tissue [14]. Mineralized bone matrix is stained in purple, collagen in pink, connective tissue in dark or light purple, osteoid in light blue, and cells as well as nuclei in blue or dark purple. It is a reliable staining in non-decalcified slices with a focus on the evaluation of bone [15]. To confirm our findings, we performed a Safranin Orange/Von Kossa staining to further specify mineralization and compared it to a non-stained slice. For further staining, we ground down the specimen and polished it again. Then, we performed a Bodian staining to detect the ingrowth of nerves. We carried out a light-microscopic evaluation of all the slices (Axio Cam MRc5, Carl Zeiss Mikroskopie, Jena, Germany).

The percentage of scaffold area and bone area in each slice was measured in ImageJ (ImageJ for java 8, version 1.52f, U. S. National Institutes of Health, Bethesda, MD, USA) using a self-implemented macro created for histological evaluations. For evaluation, tissues were identified based on image thresholding. Correct identification was individually controlled and, if necessary, manually corrected. Tissue quantification was performed automatically.

## 3. Results

**Figure 3:**
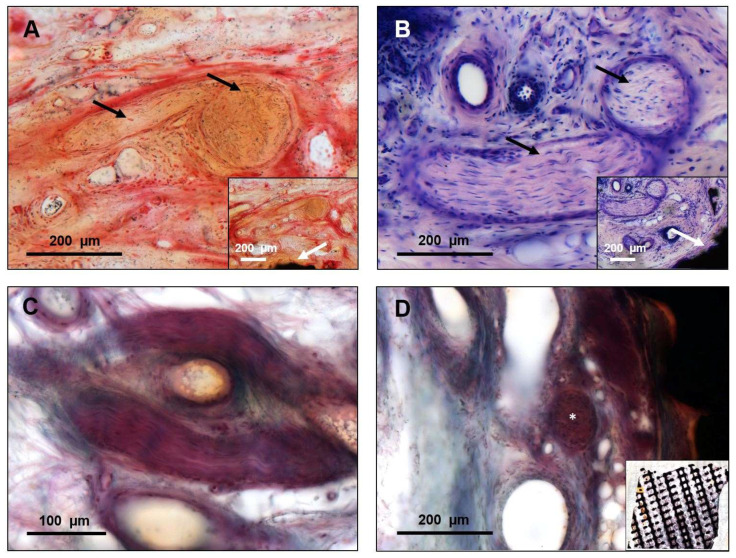
Nerve ingrowth was detected within the scaffold structure, indicating the good integration of the scaffold. (**A**) Nerves could be identified in both the Safranin O/Von Kossa staining and the Giemsa staining (**B**) outtake, indicating the proximity of the nerves to the scaffold (white arrows). (**B**) In the Giemsa staining, nerves show their typical woven structure (part 2, outer surface)—also vertically and horizontally cut (black arrow in the outtake) and the position concerning the scaffold is indicated by the white arrow in the overview. (**C**) The Bodian staining confirms that the identified structures are indeed nerves. (**D**) Overview of a Bodian staining showing the ingrowth of nerves (*) into the scaffold (part 2, outer surface).

The two frontal parts of the scaffold appeared to be filled with connective tissue (Figure 2C parts 2 and 4). At the other two partially thinner occipital parts (Figure 2C parts 1 and 3), no ingrowth at the thin areas was found. The margins were defined by surgical explantation, leading to an abrupt transition between the preserved tissue inside the scaffold and the peripheral areas. The connective tissue presented in an organized manner with fibers directly attached to the titanium, covering the complete thickness of the implant from the inner to the outer side. The vitality of the connective tissue could be demonstrated based on the existing vascularization (Figure 2G,H) and the ingrowth of nerves (Figure 3).

**Figure 4:**
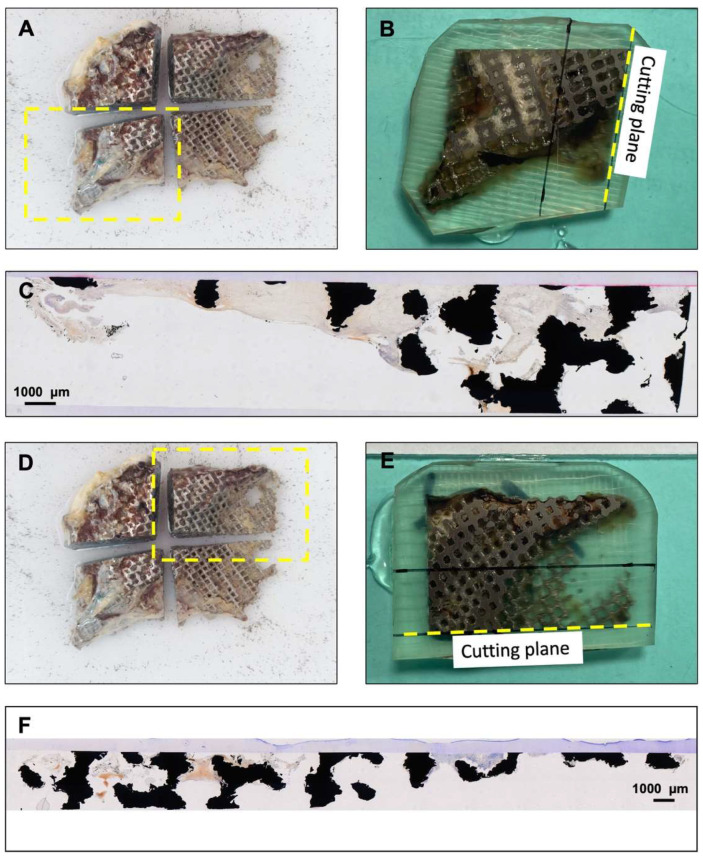
Cross sections of the scaffold confirm the lack of bone formation in the complete depth of the scaffold. Additionally, two parts, as shown in panels (**A**,**D**) were cut orthogonally to the grinding plane of the sections from the outer and inner surface. The plastic block was cut again at the marked cutting plane (**B**,**E**) and additional non-decalcified sections were prepared and stained in the Giemsa stain. Panels (**C**,**F**) show the histological sections without any signs of bone. During histological preparations, the dehydration process causes the organic tissue to shrink, but the titanium (black) stays unchanged during the embedding process. The constriction of the connective tissue is believed to be the cause of the visible gap between tissue and scaffold and the partly empty appearance of the titanium scaffold.

Despite the long period of implantation, the only exclusive bone formation was found at the bone-touching margins of the scaffold (Figure 2). All observed bone formation occurred in direct contact with titanium, but no scaffold pore was filled with woven bone. Over the whole scaffold area, mineralized bone covered 0.21% of the area of the outer surface. There was no bone detectable on the slices prepared from the inner surface. Our findings were confirmed in orthogonal cuts (Figure 4). Despite the absence of bone formation, the implant was able to provide a satisfying esthetic and functional result over several years.

## 4. Discussion

Hundreds of craniectomies are performed worldwide every day. To date, there is no standard in the reconstruction of these defects and, importantly, the reimplantation of the cryopreserved bone often fails. There is no consensus concerning the optimal alloplastic material. Complications after cranioplasty are well known and failure risks and infection rates are considered to be higher in autologous bone grafts than in alloplastic materials [4]. A recent review demonstrated significantly more reoperations for autologous bone implants than for alloplastic implants, mainly because of bone resorptions [16]. Besides lower complications, alloplastic implants allow a reduced surgery time, provide good esthetic results, and, in particular, CAD/CAM implants have advantages by reshaping complex cranial defects [17]. Some surgeons also prefer titanium implants due to lower infection rates [18]. Therefore, in this case, a CAD/CAM titanium scaffold was chosen to successfully reconstruct an esthetically important side. For many years in the postoperative course of the patient, the scaffold guaranteed a high level of mechanical integrity, protected the brain, and enabled dependable local skull stability. Overall, this underlines the high biocompatibility of titanium as a material that is known to be advantageous for such alloplastic solutions over several years [19].

Generally, biomaterials placed in bone defects should promote osteoconduction, osteoinduction, or even osteogenesis [20]. Above all, bone healing requires the presence of mesenchymal stem cells (MSCs), but it also needs a sufficient blood supply, a cascade of stimulating factors to trigger the regeneration, and the mechanical stimulation to trigger the differentiation of MSCs into osteoprogenitor cells [21].

Titanium as a material has been demonstrated to allow for osteoconduction in specific situations [22]. Recently, it was demonstrated that titanium scaffolds can enable the bone regeneration of large segmental defects in sheep and that mechano-biologically optimized scaffold designs allow an enhanced bone healing process [22]. Dynamic loading was applied in that investigation, and the construct, consisting of plate and scaffold, carried the mechanical load acting in the limb. The dependency of bone healing on mechanical stimuli is well known [23]. In this study, mechanical stimulation by the temporal muscle was presumably reduced due to detachment for surgical reasons and following complete atrophy. A further, but presumably very limited, stimulation will be permanently caused by the transmission of the heartbeat by the brain. Despite these factors, mechanical loading on skull defects is presumably almost absent or at least different compared to long bone loading.

This problem is underlined by this case, where a lack of bone overgrowth of the scaffold was found (Figure 2 and Figure 4), which resulted in direct contact of the titanium scaffold and the overlying soft tissue for many years. Except for a minimal appearance at the bone touching margins, covering 0.21% of the area of one side, there was no mineralized bone to be found inside or onside the porous structure of the scaffold. Although this is a single case, it can be assumed that CAD/CAM porous titanium scaffolds alone seem insufficient to offer enough impetus for osseointegration in such large bone defects of the skull.

It may be speculated that the covering tissue may have been irritated by the scaffold’s bare titanium surfaces and that may have led to the late infection and implant exposure in this specific case. Furthermore, the non-permanent resilience of the scalp may also be a factor [24]. Despite the absence of bone inside the scaffold, the vascularized connective tissue and nerves indicate a thorough integration into the body and a regenerative potential of the used titanium scaffold (Figure 2 and Figure 3). Although it is generally known that titanium implants may lack osseointegration in cranioplasty, this is the first study that demonstrates that titanium scaffolds in cranioplasty are well integrated into the defect in terms of soft-tissue integration.

However, this study cannot reveal if increased bone formation inside the scaffold could have prevented the late complication described in this case. Other studies have reported an increased risk for late complications in middle-aged or elderly women with surgical site infections after initial surgery [13]. Scalp skin in these patients is described as more fragile [13]. Complications might also occur due to the hardware fixing the scaffold to the bone [18]. This may also explain the findings of other studies with 17% implant extrusion [25]. In the present case, no screws or plates were used for fixation above the level of the scaffold (Figure 1G) and the exposure of the scaffold was not directly linked to the fixation. In addition, a foreign body reaction may lead to a delayed inflammatory reaction resulting in thinning of the skin and plate exposure [26]. Furthermore, *Pseudomonas aeruginosa* was found at the first detection of plate exposure. The antibiotic resistance of biofilm-enclosed bacteria on the surface of biomaterials is well known, and may have resulted in the lack of conservative treatment [27].

Besides titanium, other alloplastic materials exist for cranioplasty, of which some are in frequent clinical use. The promotion of osseointegration could also be demonstrated for carbonated calcium-based cement [28]. However, clinically their complication rate is unacceptably high and there was also no bone ingrowth into the center of the implants [28,29], therefore this material lacks in terms of advantages when compared to titanium.

As an alternative material, PEEK can also be planned and produced with CAD/CAM technology, and stands out due to its lack of imaging artifacts in comparison to titanium. Negative aspects of PEEK are its higher cost and, in particular, its lack of osteointegrative properties. [30] Clinical outcomes are limited due to the low quality of many studies and non-significant differences between PEEK and titanium [31]. Osteointegration and sufficient soft tissue coverage seems to be a major issue for long-term success, especially regarding the reason for late plate infection, as seen in this case. For comparing titanium cranioplasty versus PEEK cranioplasty, Yang et al. proposed a prospective, multicenter, non-randomized controlled trial in 2020 to evaluate the long-term outcome (trial registration number: ChiCTR2000033406) [32].

Osteointegration is also absent for PMMA, although this material is most widely used for cranioplasty [30]. Similar to PEEK, its main advantage is determined by its radiolucency, although this may result in difficulties in detecting plate fractures in the postoperative course [33]. Furthermore, PMMA is strong, reasonably cheap, and easy to use [30]. Despite these arguments, the disadvantages of PMMA include the risk of compromised esthetic results due to the necessary intraoperative hand molding and the risk of material failure when mechanical loads are applied [5,34]. Authors are also reporting on the toxicity of residual monomers, which may cause infections [35]. A main problem of PMMA is its exothermic reaction when mixed with the monomer. This may cause burn injuries of the brain and thus increase the risk of complications [36]. A possible solution to overcoming this problem is customized PMMA implants manufactured using 3D models [37]. However, within 8 years of follow-up, complications were found to occur in 23% of patients [38]. In comparison with titanium, clinical complication rates for PMMA are higher [35], indicating the generally reliable outcomes of titanium as implant material.

Overall, high complication rates of 12–50% [39] in cranioplasty emphasize the importance of improvements in reconstruction. To reduce complication rates, focus should be on the development of new materials that increase osseointegration or the mechanobiological optimization of existing materials, such as titanium. In terms of new materials, there are bioactive fiber-reinforced composite implants [40], hard-tissue-replacement implants [41], and carbon-fiber-reinforced polymers [42] described in the literature for cranioplasty. Recently, titanium plate reinforcements with calcium phosphate demonstrated promising results [43] by using the advantages of the mechanical integrity of titanium. Antibiotic loading with gentamicin, for example, is also possible in calcium phosphate implants, which might reduce clinical infections [44]. However, most alternative materials lack sufficient clinical studies [4]. Furthermore, new developments might also lose the implementation of CAD/CAM technology, which is of useful assistance for large skull defects. Hence, surface modifications and coating techniques of titanium scaffolds could represent a solution [45], and might function as an intermediate layer between the scaffold and soft tissue and thus decrease irritation and late plate exposure rates. Research should therefore also focus on techniques for the surface coating of titanium scaffolds.

## 5. Conclusions

In this study, histological analyses of a removed individual porous CAD/CAM titanium scaffold for skull reconstruction were performed for the first time. It was demonstrated that the implant provided the patient with a satisfactory result for 7.6 years. It did not support sufficient osseous integration and bony overgrowth, but vascularization and nerve infiltration were detected inside the scaffold. It remains unclear if the lack of bony overgrowth resulted in a poor long-term clinical outcome. Soft tissue infiltration inside the scaffold indicates the good biocompatibility of titanium scaffolds in general. Further research should focus on the interface between hard and soft tissue and osteoconductive surface modifications of titanium scaffolds.
